# Assessment of Liver Fat: Dual-Energy CT versus Conventional CT with and without Contrast

**DOI:** 10.3390/diagnostics12030708

**Published:** 2022-03-14

**Authors:** Jack Junchi Xu, Mikkel Ranum Boesen, Sofie Lindskov Hansen, Peter Sommer Ulriksen, Søren Holm, Lars Lönn, Kristoffer Lindskov Hansen

**Affiliations:** 1 Department of Diagnostic Radiology, Copenhagen University Hospital, Rigshospitalet, 2100 Copenhagen, Denmark; peter.sommer.ulriksen@regionh.dk (P.S.U.); lonn.lars@gmail.com (L.L.); kristoffer.lindskov.hansen.01@regionh.dk (K.L.H.); 2 Department of Clinical Medicine, University of Copenhagen, 2100 Copenhagen, Denmark; 3 Department of Clinical Physiology, Nuclear Medicine and PET, Copenhagen University Hospital, Rigshospitalet, 2100 Copenhagen, Denmark; mikkelboesen1@gmail.com (M.R.B.); sofie.lindskov.hansen@regionh.dk (S.L.H.); soeren.holm@regionh.dk (S.H.)

**Keywords:** liver fat quantification, dual-energy CT, spectral CT

## Abstract

We assessed the correlation between liver fat percentage using dual-energy CT (DECT) and Hounsfield unit (HU) measurements in contrast and non-contrast CT. This study included 177 patients in two patient groups: Group A (*n* = 125) underwent whole body non-contrast DECT and group B (*n* = 52) had a multiphasic DECT including a conventional non-contrast CT. Three regions of interest were placed on each image series, one in the left liver lobe and two in the right to measure Hounsfield Units (HU) as well as liver fat percentage. Linear regression analysis was performed for each group as well as combined. Receiver operating characteristic (ROC) curve was generated to establish the optimal fat percentage threshold value in DECT for predicting a non-contrast threshold of 40 HU correlating to moderate-severe liver steatosis. We found a strong correlation between fat percentage found with DECT and HU measured in non-contrast CT in group A and B individually (R^2^ = 0.81 and 0.86, respectively) as well as combined (R^2^ = 0.85). No significant difference was found when comparing venous and arterial phase DECT fat percentage measurements in group B (*p* = 0.67). A threshold of 10% liver fat found with DECT had 95% sensitivity and 95% specificity for the prediction of a 40 HU threshold using non-contrast CT. In conclusion, liver fat quantification using DECT shows high correlation with HU measurements independent of scan phase.

## 1. Introduction

Non-alcoholic fatty liver disease (NAFLD) is one of the most commonly encountered liver disorders worldwide [1]. NAFLD is a heterogenous disease characterized by the presence of hepatic steatosis in the absence of secondary causes such as medication or excessive alcohol consumption [2]. NAFLD is believed to be involved in the pathogenesis of Type 2 diabetes as well as cardiovascular disease and can progress to non-alcoholic steatohepatitis, fibrosis, and cirrhosis [3]. Non-invasive methods for quantifying liver fat are important in the diagnostic process and as potential tools for liver screening.

The level of hepatic steatosis associated with NAFLD can be graded with histopathology based on the fraction of hepatocytes containing fat: grade 0 (healthy, <5%), grade 1 (mild, 5–33%), grade 2 (moderate, 34–66%), and grade 3 (severe, >66%) [4]. Magnetic resonance imaging proton density fat fraction (MRI-PDFF) is considered the most precise non-invasive diagnostic tool for hepatic steatosis [5]. However, this method is costly, time consuming, and less accessible compared to other modalities capable of diagnosing hepatic steatosis, such as computed tomography (CT) and ultrasound [5]. The absolute Hounsfield Unit (HU) value of the liver in a non-contrast CT scan is predictive of liver steatosis with reported cutoffs ranging from 40–48 HU providing the diagnosis of macrovascular steatosis of 30% or greater [6,7,8]. However, CT attenuation measurements are not applicable in contrast-enhanced scans, and non-contrast CT is not considered sensitive nor specific for mild steatosis [9].

Advances in CT technology, such as Dual Energy CT (DECT), allows for qualitative and quantitative reconstructions during post-processing enabling fat percentage quantification from acquisitions with and without contrast enhancement [10,11]. We hypothesized that DECT can diagnose moderate-severe liver steatosis similarly to conventional non-contrast CT regardless of scan phase. This study assessed the correlation between liver fat percentage and HU in contrast and non-contrast-enhanced abdominal DECT scans.

## 2. Materials and Methods

### 2.1. Patients

This retrospective study was approved by the Regional Knowledge Centre on Data Protection Compliance and the Regional Committee on Health Research Ethics (P-2020-662, H-20029655). The total number of patients included in this study was 177 consisting of 2 patient groups. The first group (Group A) of 125 consecutive adult patients consisted of 71 male (73.5 ± 8.1 years) and 54 female patients (73.9 ± 7.4 years). Group A underwent non-contrast full-body DECT scan on the clinical suspicion of multiple myeloma. The second group (Group B) included 52 adult patients consisting of 23 male (63.9 ± 17.2 years) and 29 female patients (62.4 ± 15.2 years). Group B underwent a conventional non-contrast CT scan followed by a multi-phasic (arterial and venous phase) abdominal DECT scan. Indications for DECT scan performed in group B included acute gastro-intestinal bleeding/ischemia and elective characterization of renal processes.

### 2.2. DECT and Multi-Material Decomposition (MMD)

DECT utilizes two X-ray spectra of high and low energy in the image acquisition process. This allows for separation of tissue types as these express differences in the energy-dependency of attenuation across the relevant range of photon energies [12]. During post-processing, DECT allows for two and three material decomposition, meaning that the reconstruction assumes that a given region of interest (ROI) only consists of two or three materials, such as fat and water (two materials) or iodine, fat, and water (three materials). Fat contains more hydrogen atoms than other soft tissues. Therefore, the fat percentage can be mathematically quantified in each voxel based on attenuation differences derived from high and low energy photons [13,14,15,16]. A multi-material decomposition (MMD) algorithm available for research since 2013 was developed for the quantification of liver fat using fast kilovoltage (kV) switching DECT [16]. A proposed advantage of this algorithm is that it is contrast independent, which has been proven in phantoms and rabbits [17]. For non-contrast datasets, quantification is performed using two-material decomposition with fat and healthy liver as the material pair. For contrast-enhanced datasets, the algorithm applies virtual unenhancement by using a material triplet consisting of fat, blood, and contrast agent prior to a two-material decomposition as used for the non-contrast datasets.

### 2.3. Computed Tomography Acquisition

All patients were scanned in a second-generation multi-detector CT (Revolution CT ES, GE Healthcare, Milwaukee, Brookfield, WI, USA). For group A, scanning parameters were as follows: dual-energy helical scanning with 80/140 peak kV (kVp) fast switching with an automatic exposure control (GSI Assist, GE Healthcare, Milwaukee, WI, USA) and a noise index of 13, rotation time of 0.5 s, pitch of 0.992, slice thickness of 3 mm, and beam width of 80 mm [18]. For group B, conventional non-contrast images were acquired with 120 kVp, AutomA function (range: 80–600 mA) with a noise index of either 13 or 18, rotation time of 0.5 s, helical pitch of 0.992, slice thickness of 3 mm, and beam width of 80 mm. Dual-energy scan parameters for group B were identical to group A aside from the mA mode, which was set to 200 mA with GSI Assist.

Omnipaque 350 mg/mL (Iohexol, GE Healthcare, Oslo, Norway) was used for the contrast-enhanced DECT scans. Contrast was administered through an 18-gauge plastic cannula in the antecubital vein. Contrast volume was either 100 mL or 130 mL for a patient between 60–90 kg at a flowrate of 4 mL/s. The delay for arterial and venous phase was either 7 s or 15 s and 45 s or 55 s, respectively, following HU threshold triggering within the aorta set to 100 and 120 HU.

### 2.4. Image Analysis

**Figure 1:**
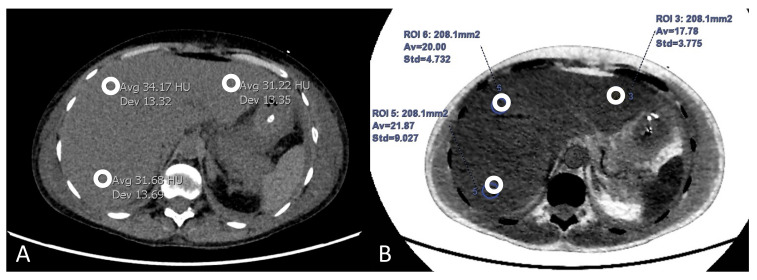
An example of measurements from ROIs placed at three designated liver positions in the left and right liver lobe within the same patient. (**A**) The HU measurements for a conventional non-contrast CT scan. (**B**) The corresponding fat percentage measurements made within the AW software on an arterial phase reconstruction. The ROIs in (**A**,**B**) are marked with white circles.

The attenuation HU measurements were conducted using Impax PACS (Version 6.7.0.6011, Agfa Healthcare, Mortsel, Belgium). Fat percentage measurements for non-contrast (Group A) as well as arterial and venous phase (Group B) scans were made using the liver fat algorithm within the Advanced Workstation (AW) software (GE Healthcare, Waukesha, WI, USA). For group A, HU measurements using ROIs were performed on a non-contrast virtual monoenergetic reconstruction at 74 keV, whereas HU measurements were performed on the conventional 120 kVp image series, corresponding to the effective energy of the monoenergetic reconstruction of 74 keV for group B [19]. Hence, fat percentage was calculated from DECT and compared to HU measurements from non-enhanced CT in both groups. In group A, the non-enhanced CT images were obtained with DECT reconstruction at 74 keV, while conventional 120 kVp images were used in group B. Three ROIs were placed according to a defined modification of the Couinaud hepatic segmentation system (Figure 1) [20]. One ROI was placed in the left liver lobe (segment II or III), and two ROIs were placed in the right liver lobe labeled right lobe 1 (segment VII or VIII) and right lobe 2 (segment V or VI). ROIs were carefully placed avoiding large vessels and focal lesions at either the exact (group A) or approximated position (Group B). Each ROI measured 2.00 ± 0.15 cm2, and measurements were conducted by a second-year radiology resident. HU and fat ratio measurements were conducted separately to blind the observer from the referencing data.

### 2.5. Statistical Analysis

Data were analyzed using MATLAB (MathWorks, Version R2020b, Natick, MA, USA). Data were initially analyzed with descriptive statistics, i.e., means and standard deviations. Fat percentage and HU were averaged for each patient, and the correlation with confidence intervals (CI) between fat percentage and HU measurement was compared using linear regression analysis. This was performed separately for the non-contrast- and contrast-enhanced image data and residual variance was calculated for the combined datasets. The receiver operating characteristic (ROC) curve was generated to establish the optimal threshold value for fat percentage for predicting a non-contrast threshold of 40 HU.

## 3. Results

The overall mean fat percentage for all patients was 5.8 ± 9.7% with a median fat percentage of 2.9%, corresponding to a mean attenuation of 54.5 ± 12.5 HU with a median of 57.7 HU. For group A, the mean fat percentage was 5.0 ± 6.5% corresponding to 55.9 ± 11.0 HU. For group B, the mean fat percentage was 7.5 ± 9.7% with a mean attenuation of 51.2 ± 15.2 HU. There was no significant difference between group A and B for mean fat percentage or mean attenuation; *p* = 0.30 and *p* = 0.39, respectively.

**Figure 2:**
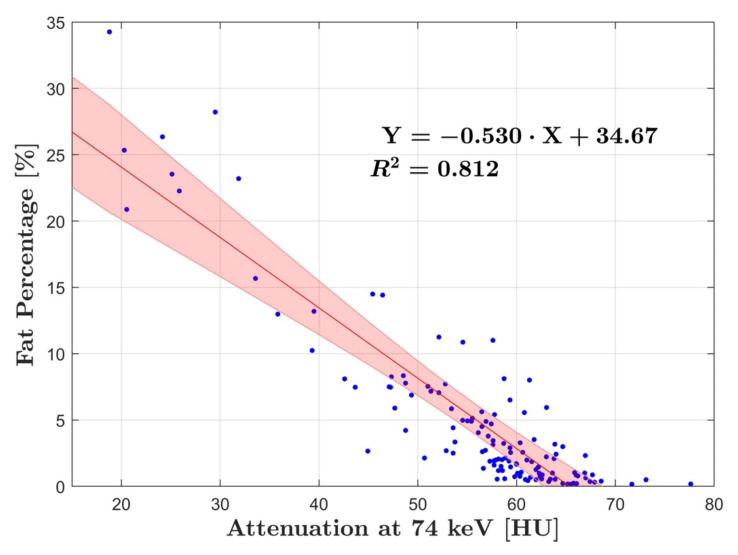
Linear regression analysis of group A showing the relationship between fat percentage and HU at 74 keV. The analysis showed strong correlation with an R^2^ of 0.81. The blue dots represent the average fat and HU measurements for each patient, while the red line corresponds to the line of best fit, with the red shaded area showing the 95% CI.

**Figure 3:**
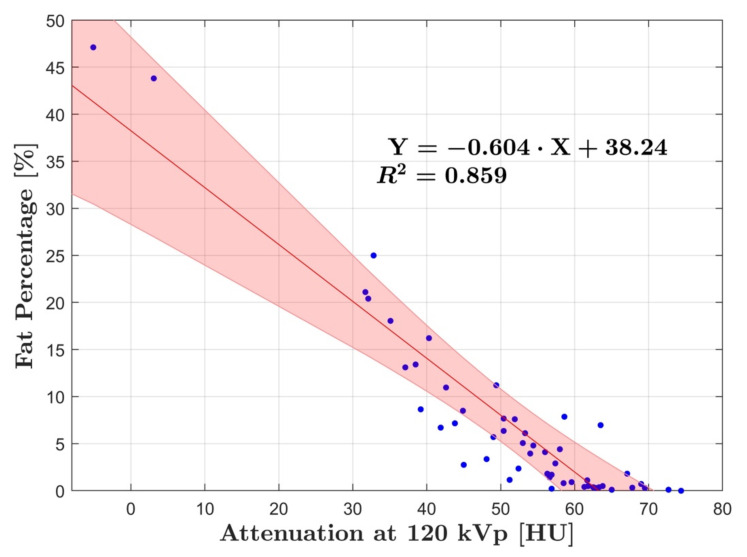
Linear regression analysis of group B showing the relationship between fat percentage and. HU at 120 kVp. Strong correlation was found R^2^ = 0.86. The blue dots represent the average fat and HU measurements for each patient, while the red line corresponds to the line of best fit, with the red shaded area representing the 95% CI.

**Figure 4:**
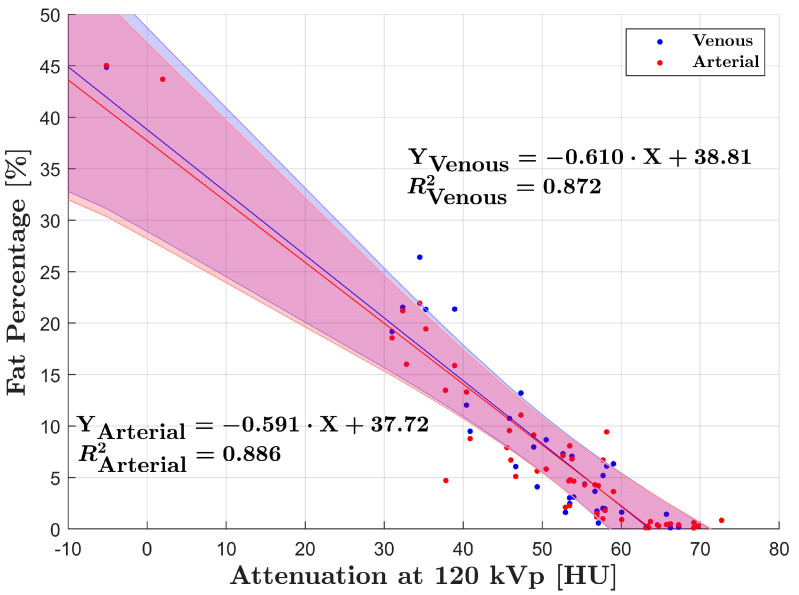
Linear regression analysis of arterial and venous data points for group B. Strong correlations were found for both linear regressions R^2^ = 0.87 (venous) and 0.89 (arterial). The red and blue lines correspond to line of best fit for each analysis. The overlapping shaded areas represent the 95% CI for each linear regression.

Linear regression analysis of fat percentage compared to HU in group A and B showed strong correlation with R^2^ = 0.81 (*p* < 0.001) and R^2^ = 0.86 (*p* < 0.001), respectively (Figure 2 and Figure 3). DECT fat percentage measurements in group B were averaged as arterial and venous datasets demonstrated no significant difference (*p* = 0.67). Additionally, correlation of fat percentage and HU for the arterial and venous phase individually was R^2^ = 0.89 (*p* < 0.001) and R^2^ = 0.87 (*p* < 0.001), respectively (Figure 4).

**Figure 5:**
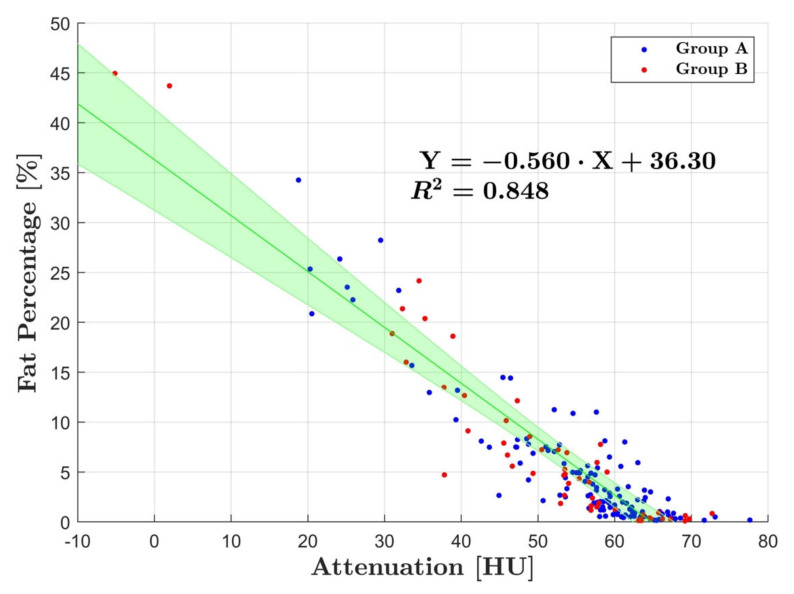
Linear regression analysis with group A and B combined. There was a strong correlation between fat percentage and HU with R^2^ = 0.85. The blue dots represent the average measurements for group A and the red for group B. The green line corresponds to line for best fit and the shaded area represents the 95% CI.

The slope defined by regression lines in group A and B was −0.60 ± 0.06 and −0.53 ± 0.05, respectively. When combining the data from group A and B, in which arterial and venous fat percentage measurements in group B were averaged, a strong correlation to HU was calculated of R^2^ = 0.85 (*p* < 0.001) with a slope of −0.56 and a residual variance of ± 5.89% (Figure 5).

**Figure 6:**
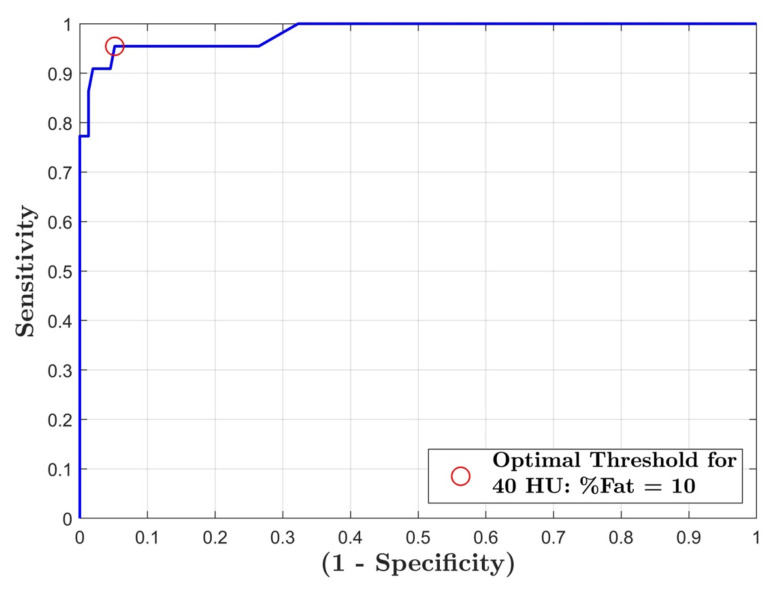
ROC curve for fat percentage threshold with a HU cut-off of 40 HU. Area under the curve (AUC) = 0.98.

The area under the ROC curve was 0.98 (Figure 6). A threshold of 10% liver fat had 95% sensitivity and specificity for predicting a non-contrast threshold of 40 HU corresponding to moderate-severe hepatic steatosis. Linear regression analysis for each individual ROI measurement showed that two of nine possible comparisons were statistically significant (Table 1), these being group A left lobe vs. group A right lobe 1 (*p* = 0.04) and group A left lobe vs. group B left lobe (*p* = 0.003).

## 4. Discussion

This study showed an overall significant correlation between fat percentage and HU measurements using fast kV-switching DECT independent of scan phase. A threshold of 10% liver fat using DECT is highly sensitive and specific in predicting a non-contrast liver attenuation threshold of 40 HU, utilizing non-contrast CT corresponding to moderate-severe liver steatosis.

Existing literature indicates that a liver attenuation <40 HU on a non-contrast CT scan corresponds to a moderate-severe liver steatosis or a liver fat percentage of ≥30% [6,21]. In this study, 10% fat content employing DECT corresponded to 40 HU (Figure 6). The difference in threshold found in this study compared to the literature is likely due to the methodology of the histopathological reference standard, which assesses the fat percentage based on the fraction of hepatocytes with fatty vesicles [22,23]. A histopathologist may in theory report 100% steatosis based on the premise that 100% of hepatocytes are involved with steatosis, while fat quantification using MRI-PDFF or DECT will report a substantially lower fat content due to water protons and soft-tissue present in the ROI. This was demonstrated in studies correlating MRI-PDFF and histopathological steatosis grading with lower MRI acquired cut-offs of 16.3% and 21.7%, corresponding to histopathological steatosis grading of 1–2 and 2–3 [24,25]. A similar relationship between DECT fat percentage measurements and histopathological may explain the threshold found in this study. Therefore, a conversion factor may aid in the prediction of the histopathological fat fraction based on fat quantification using DECT.

The diagnosis of hepatic steatosis is challenging in contrast-enhanced CT imaging as liver attenuation is altered, and liver-minus-spleen attenuation differences vary due to contrast injection rates and timing of measurements [26,27]. In these cases, liver fat quantification using MMD can be an alternative, as it is unaffected by contrast phase. The comparison of the linear regression models for the different liver segments showed that the left lobe of group A was significantly different from the left lobe in group B as well as right lobe 1 in group A. Aside from the differences between the two populations, technical factors such as varying image noise within the field of view may increase variability in the measurements especially in obese patients [28]. Additionally, previous studies using MRI-PDFF and MR spectroscopy (MRS) have shown differences in fat distribution within the liver lobes in non-diabetic patients and NAFLD patients [29,30]. These studies suggested a slightly lower fat percentage in the left liver lobe, albeit with a significantly higher variability (*p* < 0.0001) [30]. These factors may explain the significant differences found in this study and should be considered in future studies when selecting ROI for data analysis.

To our knowledge, no studies have compared liver fat quantification using this MMD algorithm with HU measurements. One study by Patel et al. assessed the relationship between HU and material density fat (-iodine) in 363 consecutive adult patients undergoing multiphasic abdominal DECT [31]. In this study, a high correlation was found between HU and fat (-iodine) measurements (R^2^ = 0.74) with a sensitivity, specificity, and area under the curve (AUC) of 71%, 80%, and 0.85, respectively. However, several differences separate this study from the study by Patel et al. First, our data suggest that DECT liver fat quantification has an even higher correlation to HU and is highly sensitive and specific for the diagnosis of moderate-severe hepatic steatosis when compared to HU. This may be due to a more specific and improved algorithm for liver fat quantification. Secondly, this study used a newer scanner enabling an increased energy separation of 20%, which may also explain why we found an increased correlation as well as improved sensitivity and specificity [32].

Further studies to evaluate liver fat content in patients with varying degrees of steatosis (mild to severe) using DECT and either MR-PDFF or histopathological findings as reference, are warranted. In a study by Hyodo et al., DECT was accurate and reproducible in diagnosing liver steatosis compared to MRS and histopathology across scan phases [16]. In this study, both MRS and DECT showed increasing fat fraction with increasing histopathologic steatosis grading, and ROC analyses for discrimination between grade 0 and grade 1–3 yielded an AUC of 0.88 and 0.89 for DECT and MRS, respectively.

This study has several limitations. The non-specific study populations with limited data points especially for severe steatosis influenced the slope and cut-off for the linear regression groups. Furthermore, MRI and/or biopsy assessments for liver fat content would have provided an improved reference compared to CT attenuation, which primarily has been shown to be sensitive in diagnosing moderate-severe steatosis [7,8].

Future studies should evaluate the reproducibility of DECT prior to clinical implementation. Lastly, increased iron content within the liver may affect the assessment of fat quantification and warrants further investigation [33].

## 5. Conclusions

Our study identified strong correlation between HU and liver fat percentage measurements using fast kV-switch DECT and a dedicated MMD liver fat quantification algorithm regardless of scan phase. A threshold of 10% liver fat using DECT resulted in a sensitivity and specificity of 95% for prediction of a 40 HU threshold indicating moderate-severe liver steatosis. Liver fat quantification can be utilized in the presence of contrast media and can be an alternative method for moderate-severe liver steatosis diagnosis in both non-contrast- and contrast-enhanced DECT scans.

## Figures and Tables

**Table 1:** *p*-values derived from the comparison of different liver segment linear regression analyses. Significant *p*-values (*p* < 0.05) are denoted by ^§^. * denotes comparison between group A and B, ** within group A, and *** within group B.

	Left Lobe	Right Lobe 1	Right Lobe 2
Left lobe	0.003 *^§^	0.04 **^§^	0.43 **
Right lobe 1	0.81 ***	0.19 *	0.67 ***
Right lobe 2	0.48 ***	0.23 **	0.08 *

## Data Availability

The data that support the findings of this study are available from the corresponding author (J.J.X.), upon reasonable request.
